# Ocrelizumab in early relapsing–remitting multiple sclerosis: first interim analysis of the MUSPO Italian prospective cohort

**DOI:** 10.1007/s00415-026-13830-0

**Published:** 2026-05-04

**Authors:** Massimo Filippi, Emanuele D’Amico, Vincenzo Brescia Morra, Chiara Zanetta, Aurora Zanghì, Paola Sofia Di Filippo, Elena Longobardi, Elena Colombo, Massimiliano Mirabella, Antonella Conte, Valentina Tomassini, Cinzia Masini, Valeria Perosino, Paola Tortorella, Maria Assunta Rocca

**Affiliations:** 1 Neurology Unit, IRCCS San Raffaele Scientific Institute, Via Olgettina, 60 - 20132 Milan, Italy; 2 Neuroimaging Research Unit, Division of Neuroscience, IRCCS San Raffaele Scientific Institute, Milan, Italy; 3 Neurorehabilitation Unit, IRCCS San Raffaele Scientific Institute, Milan, Italy; 4 Neurophysiology Service, IRCCS San Raffaele Scientific Institute, Milan, Italy; 5 Vita-Salute San Raffaele University, Milan, Italy; 6 Department of Medical and Surgical Sciences, BRAND, University of Foggia, Foggia, Italy; 7 Department of Neuroscience, Reproductive Science and Odontostomatology, Federico II University of Naples, Naples, Italy; 8 Multiple Sclerosis Unit, Policlinico Federico II University Hospital, Naples, Italy; 9 Multiple Sclerosis Centre, IRCCS Mondino Foundation, Pavia, Italy; 10 UOC Di Neurologia, Fondazione Policlinico Universitario “A. Gemelli” IRCCS, Rome, Italy; 11 Department of Human Neurosciences, Sapienza University of Rome, Rome, Italy; 12 IRCCS Neuromed, Pozzilli, Italy; 13 Centre for Disability, Rehabilitation and Sport Medicine (CARES) & Institute for Advanced Biomedical Technologies (ITAB), Department of Neurosciences, Imaging and Clinical Sciences, University G. d’Annunzio, Chieti, Italy; 14 Roche SpA, Monza, Italy

**Keywords:** Ocrelizumab, Real-world evidence, NEDA-plus, RRMS, RES

## Abstract

**Background:**

Real-world evidence on ocrelizumab in early, minimally pretreated relapsing multiple sclerosis (MS) patients remains limited.

**Objective:**

To describe baseline demographic, clinical, radiological, and treatment characteristics of the MUSPO cohort.

**Methods:**

MUSPO is a prospective, multicenter, observational study. Enrollment occurred from September 2023 to May 2025. Adults with relapsing–remitting MS (RRMS), including a rapidly evolving severe RRMS (RES) group, were enrolled ~ 6 months after starting ocrelizumab. MRI was acquired according to clinical practice with centralized reading. NEDA-plus (absence of relapses, disability worsening, MRI activity, brain atrophy, and cognitive worsening) will be assessed at years 1–4. Baseline descriptive statistics were used.

**Results:**

Of 208 patients enrolled across 31 Italian sites, 199 were eligible (RRMS N = 59; RES N = 140) for the interim analysis. Median Expanded Disability Status Scale was 2.0 (IQR 1.0–3.0), indicating mild disability. Prior disease-modifying therapy (DMT) exposure occurred in 59/199 patients (29.6%); time from last DMT to ocrelizumab was a median 1.4 months (IQR 0.7–2.0). MRI at diagnosis was available for 188/199 patients (94.5%), gadolinium used in 157/188 scans (83.5%). Baseline MRI was available for all patients with gadolinium administered in 166/199 cases (83.4%). Cognitive assessment using the Symbol Digit Modalities Test was completed by 150/199 (75.4%) of patients.

**Conclusions:**

The MUSPO study captures a predominantly young, mildly disabled, early-treated cohort, some of whom have prior DMT exposure. Together with comprehensive baseline MRI and limited prior DMT use, these features provide a robust foundation to evaluate NEDA-plus and other longitudinal effectiveness endpoints in Italian practice.

**Supplementary Information:**

The online version contains supplementary material available at 10.1007/s00415-026-13830-0.

## Introduction

Multiple sclerosis (MS) is a chronic, immune-mediated, demyelinating disease of the central nervous system characterized by inflammation, demyelination, axonal loss, and progressive neurological dysfunction [1]. It represents one of the leading causes of non-traumatic neurological disability in young adults, particularly affecting women in their second to fourth decades of life [2]. Globally, more than 2.8 million individuals have MS, corresponding to a prevalence of ~ 36 cases/100,000 individuals (or ~ 0.036%) [3]. In Italy, prevalence ranges between 180—250/100,000 individuals, with approximately 130,000 people living with the disease [3].

Relapsing–remitting multiple sclerosis (RRMS) accounts for nearly 85% of initial diagnoses [4]. The accumulation of irreversible disability in MS is primarily attributed to neuroaxonal loss (neurodegeneration), a process occurring as a consequence (but not exclusively) of focal demyelination and diffuse, persistent inflammation, with both aspects measurable subclinically via magnetic resonance imaging (MRI) [5]. Growing evidence shows that initiating high-efficacy therapy early and maintaining it over time can limit disease activity and reduce the risk of future disability progression. As a result, current treatment guidelines increasingly recommend this strategy from the earliest stages of relapsing MS [3, 6].

Over the past two decades, the treatment for MS has evolved considerably. The first-generation injectable agents offered moderate efficacy in reducing relapse rates and MRI activity but limited effects on disability progression [7]. Subsequent oral therapies provided greater convenience and improved efficacy in selected patients [8]. More recently, high-efficacy monoclonal antibodies targeting T-cell trafficking or B cells have redefined treatment strategies, enabling earlier disease control and potentially improved long-term outcomes [9]. Among these, ocrelizumab, a humanized anti-CD20 monoclonal antibody, has shown robust efficacy in controlling both clinical relapses and MRI activity [10, 11], as demonstrated in the pivotal OPERA I and II trials [12, 13], with pooled analyses confirming sustained suppression of disease activity and brain volume loss [14]. Long-term ocrelizumab data indicate greater reductions in disability progression when treatment is initiated early, together with a favorable long-term safety profile [14]. Consistent findings also emerge from the ENSEMBLE study, which evaluated ocrelizumab in treatment-naïve patients with early-stage RRMS [15].

Beyond clinical trials [16], real-world studies have provided complementary insights into ocrelizumab’s effectiveness and safety [17]. However, most real-world cohorts have included patients with multiple prior disease-modifying therapies (DMTs), with limited data on individuals starting ocrelizumab early in their disease course or close to the diagnosis. Furthermore, standardized evaluation of cognitive function, MRI measures of brain atrophy, and patient-reported outcomes (PROs) remains limited and with short follow-up in observational settings [17].

Given the need for complete disease control, the concept of no evidence of disease activity (NEDA) has evolved to include brain volume loss and cognitive worsening, defining the NEDA-plus framework as the aspirational therapeutic goal [18, 19]. Real-world studies addressing the attainment of NEDA-plus in patients treated with ocrelizumab, particularly those initiating therapy early or following a suboptimal response to a single prior DMT, are limited [20].

The MUSPO study (The evaluation of relapsing–remitting MUltiple Sclerosis progression in Patients taking Ocrelizumab in a prospective observational study: the MUSPO cohort Italian study) was designed for addressing this gap. The study enrolled Italian patients with RRMS treated with ocrelizumab in a real-world setting, focusing specifically on cohorts underrepresented in current real-world evidence (i.e., suboptimal responders to one previous DMT and patients with rapidly evolving severe RRMS, as defined by specific clinical and MRI criteria of the label, hereafter referred to as RES). MUSPO aims to define the effectiveness of ocrelizumab by assessing the proportion of patients achieving NEDA-plus over four years, integrating comprehensive clinical, MRI, and cognitive assessments. This paper describes the first interim analysis which aims to characterize the MUSPO cohort at baseline, providing a reference framework, essential for future longitudinal effectiveness results.

## Materials and methods

### Study design and setting

This is a prospective, multicenter, observational study conducted under routine clinical practice to evaluate the effectiveness of ocrelizumab in MS. Patient recruitment was completed on May 19, 2025; therefore, the study cohort is closed and no additional patients will be enrolled. Patients will be followed for up to four years from enrollment, which occurred approximately six months after treatment initiation (after the two initial 300 mg infusions and before the first 600 mg infusion). Study follow-up will be completed by May 19, 2029, according to the study protocol.

Approximately 200 eligible patients were planned to be enrolled. Given the non-interventional and uncontrolled design, the sample size was based on feasibility considerations rather than formal statistical power calculations, in line with the descriptive and exploratory objectives of the study. No randomization or control arm was included. All clinical decisions, including the choice to treat with ocrelizumab over alternative B-cell therapies or other DMT, were made by the investigators in accordance with clinical practice, the current Summary of Product Characteristics (SmPC), and local labeling. The specific reasons underlying treatment selection were not systematically collected. Study participation did not alter standard care.

Two protocol versions were in use during enrollment (v1.0 and v2.0), differing only in the definition of inclusion criterion D, which sets the allowable diagnostic time window for eligibility (≤ 2 years in v1.0 vs ≤ 5 years in v2.0 for patients meeting Criterion #1). Full details are provided in Supplementary Table S1.

### Participants

Adults (≥ 18 years) with a definite diagnosis of RRMS according to 2017 McDonald criteria [21] and an Expanded Disability Status Scale (EDSS) score ≤ 3.5 were eligible if they had started ocrelizumab as part of routine practice approximately six months before enrollment and had not yet received the second 600 mg (third overall) infusion. Two prespecified groups were defined, corresponding to the eligibility categories established by the Italian Medicines Agency (AIFA) reimbursement label. The first comprised patients with inadequate response to one prior DMT, defined by at least one relapse in the previous year while on therapy together with at least one new/enlarged T2-hyperintense or gadolinium-enhancing (Gd +) lesion on MRI; patients who had discontinued the prior DMT for safety or tolerability (e.g., adverse events or other clinical considerations in accordance with local labeling) were also eligible. The second comprised patients with RES, defined by at least two disabling relapses in one year together with at least one new Gd + lesion or a marked increase in T2-hyperintense lesion burden on brain MRI. The diagnosis of RRMS had been established within five years before study entry for the first group and within two years for the second. All participants provided written informed consent and were able and willing to complete PROs.

Patients were excluded if they were not receiving ocrelizumab for RRMS according to standard of care or approved labeling, or if they had been previously treated with anti-CD20 monoclonal antibodies. Information collected from enrolled patients included lifestyle habits, general medical history information and disease history.

### Treatment and assessments

Ocrelizumab was administered per routine practice (two 300 mg infusions two weeks apart, followed by 600 mg infusions every six months). All clinical assessments were conducted according to routine clinical practice. Protocol-defined relapses, EDSS scores, and concomitant medications were collected at baseline and at annual protocol-specified follow-up visits. Cognitive function was primarily evaluated using the Symbol Digit Modalities Test (SDMT), corrected as described by Amato et al. [22], which serves as the cognitive component of the NEDA-plus endpoint. The short Rao’s Brief Repeatable Battery (BRB-N) was also collected for exploratory cognitive analyses [22]. PROs included the Multiple Sclerosis Impact Scale (MSIS-29) [23], the Modified Fatigue Impact Scale (MFIS) [24], the Treatment Satisfaction Questionnaire for Medication (TSQM) [25], and the Multiple Sclerosis Cost of Illness (MS-COI) [26] questionnaire. They were self-administered at baseline and annually via a web platform, with completion allowed on the day of drug administration or up to five weeks earlier; paper forms were used when electronic completion was not available.

### MRI acquisition and analysis

MRI assessments followed clinical practice at sites, in accordance with MAGNIMS (Magnetic Resonance Imaging in Multiple Sclerosis, www.magnims.eu) recommendations [27]. Acquisitions were performed by using both 1.5 T and 3.0 T scanners; scanner/protocol changes were possible (but not recommended) between patients of the same center. Structured electronic capture of local MRI reading included the following metrics: 1) brain/spinal cord Gd + and T2-hyperintense lesion count on MRI performed at diagnosis, 2) brain/spinal cord Gd + lesion count and T2-hyperintense lesion count on baseline MRI, and 3) new/enlarged T2-hyperintense and Gd + lesions relative to the last available scan prior to baseline. Baseline MRI data were also sent to the Neuroimaging Research Unit, IRCCS Ospedale San Raffaele (Milan, Italy) through a secure and anonymized electronic interface to undergo centralized reading. Among the received sequences, the following ones were selected for the assessment of centralized MRI measures: 1) dual-echo (DE) turbo spin echo (TSE) or 3D/2D fluid-attenuated inversion recovery (FLAIR) for calculation of T2-hyperintense lesion volume (LV); 2) 3D/2D post-contrast T1-weighted scans for Gd + lesion count; 3) 2D pre- or post-contrast T1-weighted TSE for calculation of T1-hypointense LV. Whenever available, the 3D pre-contrast T1-weighted sequence was also selected for the longitudinal calculation of brain and upper cervical cord atrophy (needed for NEDA assessments). When an MRI at study entry was unavailable, the last pre-enrollment MRI performed within the preceding 12 months was used as baseline for longitudinal evaluations.

The centralized MRI analysis consisted in the identification and marking, performed by one trained operator (supervised by a senior operator), of T2-hyperintense white matter lesions on DE/FLAIR scans, of Gd + lesions on post-contrast T1-weighted scans, and of T1-hypointense lesions on T1-weighted TSE scans. Gd + lesions were counted, while T2-hyperintense and T1-hypointense LVs were measured by a trained Technician with a local thresholding segmentation technique (Jim 8.0 software; Xinapse Systems, Colchester, UK).

### Study outcomes

The primary effectiveness outcome is the proportion of patients having NEDA-plus [19] at year 4, defined by the absence of relapses, no 1-year EDSS-confirmed disability progression, no MRI activity (at least one new Gd + lesion or a new/enlarged T2-hyperintense lesion), no brain volume loss (as assessed by local MRI reading), and no cognitive worsening at the SDMT. NEDA-plus will also be assessed at years 1, 2, and 3. Secondary outcomes will include NEDA-plus based on centralized MRI evaluations, change in cervical cord area based on centralized MRI evaluation, and annual trajectories of PROs. Additional analyses will include employment status and healthcare resource use. Safety objectives comprise the description and frequency of adverse events (AEs), serious AEs (SAEs), and adverse events of special interest (AESIs), collected according to Good Clinical Practice (GCP) and categorized by system organ class and severity.

Exploratory objectives include extended cognitive assessments based on the BRB-N [22], and exploratory definitions of NEDA-plus incorporating additional MRI-derived measures, such as centrally assessed spinal cord atrophy (i.e., cervical cord cross-sectional area), alongside percentage brain volume change.

### Optional immune sub-study

Participation in the immunological sub-study was limited to centers with the appropriate infrastructure and logistical capacity for biological sample collection, processing, and storage. A consenting subgroup of patients (N = 72) underwent high-resolution, multiparametric flow-cytometry profiling of lymphocyte subsets at six-month intervals aligned with ocrelizumab infusions. Blood samples were collected in EDTA tubes, processed locally, and peripheral blood mononuclear cells (PBMCs) and plasma were cryopreserved before being shipped to a central laboratory for analysis. Immune profiling included the assessment of T-cell and B-cell phenotypes, cytotoxic markers, and indices of immune senescence.

### Data management, ethics, and safety

Data were recorded in an electronic case report form (eCRF) implemented on the Actide platform (Nubilaria). Clinical data were entered by treating clinicians, while PROs were self-administered via a web-based platform or entered into the eCRF by clinicians when collected on paper. The database for this first interim analysis was locked on 19 May 2025 (data cut-off).

This study was conducted in compliance with the Declaration of Helsinki, GCP standards, International Council for Harmonisation (ICH) guidelines, and national regulations. Ethical approval was granted on 31 May 2023 from the Territorial Ethics Committee – South West Veneto (Comitato Etico Territoriale Area Sud Ovest Veneto) by the institutional ethics committee (protocol ID: ML44477), and all participants provided informed consent. Safety information was collected according to routine practice, with procedures in place for follow-up of adverse events, product complaints, pregnancies, and medication errors.

### Statistical analysis

In this first interim analysis, data are descriptive and focused on baseline characterization of the eligible population. Categorical variables were summarized as counts and percentages (including valid percentages when applicable), and continuous variables as means with standard deviations or medians with interquartile range (IQR). Analyses were performed using IBM SPSS Statistics (version 27).

## Results

### Study population: enrollment and eligibility

Between 29th September 2023 and 19th May 2025, 208 patients were enrolled at 31 sites (Supplementary Table S2). Eligibility was assessed according to the predefined inclusion and exclusion criteria (Supplementary Table S1). Enrollment occurred under protocol version v1.0 in 41 patients (19.7%) and under v2.0 in 167 (80.3%) (Supplementary Table S2). Site-level accrual ranged from 1 to 22 patients; the full site-by-version distribution is presented in Supplementary Table S3.

**Fig. 1:**
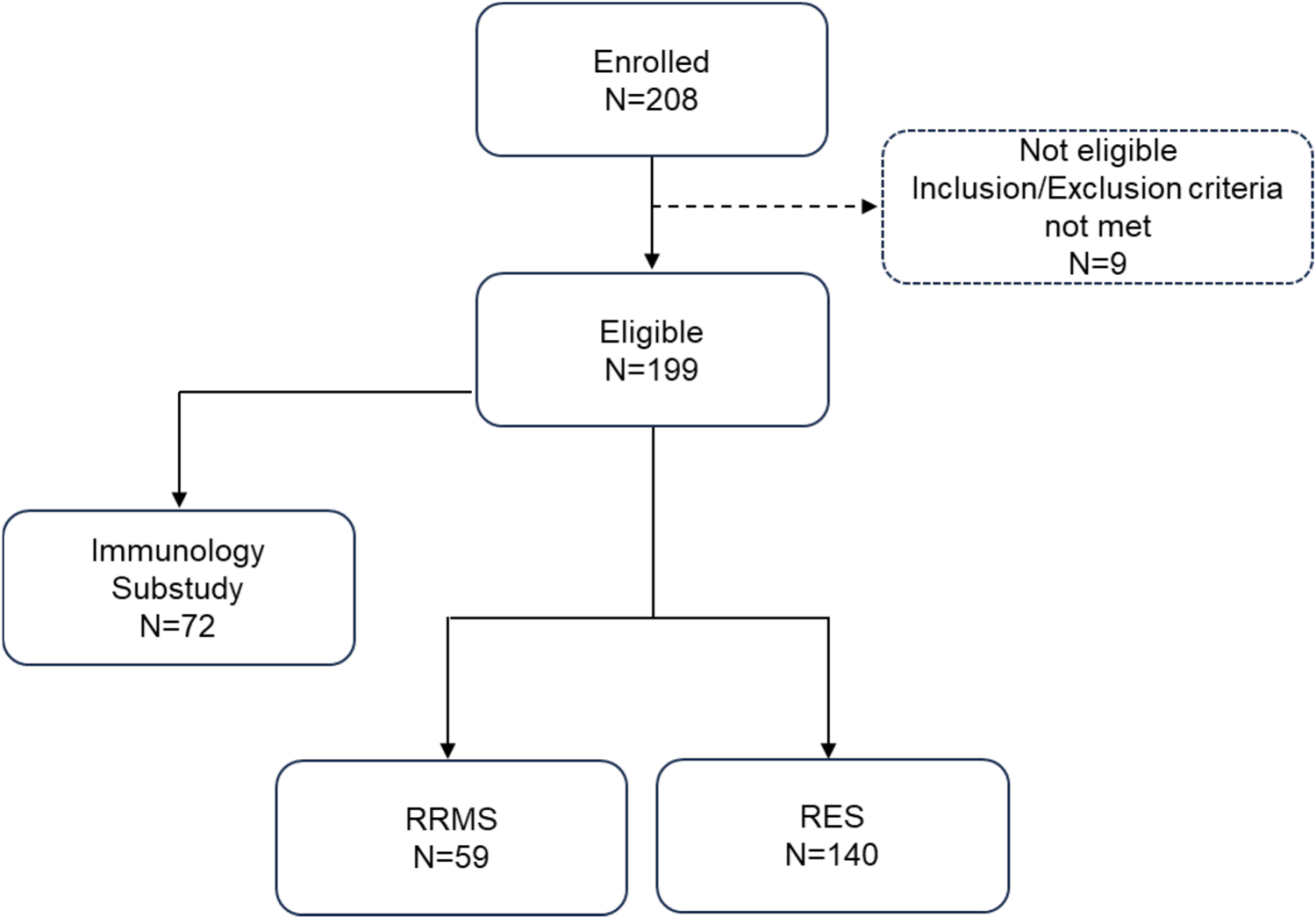
Patient disposition. Of 208 enrolled patients, 199 met eligibility criteria and were included in the overall population for analysis, while 9 were ineligible. Among eligible patients, 72 entered the optional immunology sub-study. The overall population comprised two clinical groups: RRMS with inadequate response to one prior disease-modifying therapy, DMT (N = 59), and rapidly evolving severe multiple sclerosis (RES) (N = 140). *RRMS* relapsing–remitting multiple sclerosis; *RES* rapidly evolving severe sclerosis

A total of 199 (95.7%) met eligibility and were included in the analysis, with 59 (29.6%) classified as RRMS and 140 (70.4%) as RES (Fig. 1). Regarding protocol version in the analysis set, 39 (19.6%) were enrolled under v1.0 and 160 (80.4%) under v2.0 (Supplementary table S2).

### Baseline characteristics

Baseline characteristics of the eligible population (N = 199) are summarized in Table 1. Mean age at enrollment was 36.5 ± 11.3 years, with the < 40 years age category being the most represented (62.3% overall; 31.5% RRMS; 68.5% RES). Females accounted for 114 of all participants (57.3%), while males accounted for 85 (42.7%), with a different distribution between groups (30.7% vs 69.3% for females in RRMS and RES, respectively).

**Table 1:** Baseline demographic and disease characteristics in the overall population and by groups

Characteristic	Total (N = 199)	RRMS (N = 59)	RES (N = 140)
Age, mean (SD)	36.5 (11.3)	36.4 (10.5)	36.6 (11.6)
Age, n (%)			
< 40 years	124 (62.3)	39 (31.5)	85 (68.5)
40–55 years	63 (31.7)	17 (27.0)	46 (73.0)
> 55 years	12 (6.0)	3 (25.0)	9 (75.0)
Sex, n (%)			
Male	85 (42.7)	24 (28.2)	61 (71.8)
Female	114 (57.3)	35 (30.7)	79 (69.3)
BMI, kg/m^2^			
N (missing)	189 (10)	57 (2)	132 (8)
Mean (SD)	24.4 (4.5)	25.8 (5.6)	23.8 (3.8)
Smoking, n (%)			
Non-smoker	122 (61.3)	36 (29.5)	86 (70.5)
Cigarettes	53 (26.6)	18 (34.0)	35 (66.0)
Device/e-cigarettes	9 (4.5)	2 (22.2)	7 (77.8)
Past smoker	15 (7.5)	3 (20.0)	12 (80.0)
Alcohol, n (%)			
Occasional	116 (58.3)	36 (31.0)	80 (69,0)
Never or none in last 12 months	81 (40.7)	23 (28.4)	58 (71,6)
Daily	2 (1.0)	0 (0.0)	2 (100)
EDSS score, median (IQR)	2.0 (1.0—3.0)	2.0 (1.0—2.5)	2.0 (1.0—3.0)

Data refer to the eligible set (N = 199) unless otherwise specified

Data are reported as numbers (% of category total) or median (IQR)

*RRMS* relapsing–remitting multiple sclerosis; *RES* rapidly evolving severe relapsing–remitting multiple sclerosis; *BMI* body mass index; *IQR* interquartile range (Q1–Q3); *N*: number of patients with available data

Mean body mass index (BMI) was 24.4 ± 4.5 kg/m^2^, with comparable values across groups.

Regarding lifestyle habits, non-smoking status was reported in 122 patients (61.3%), 36 (29.5%) among RRMS and 86 (70.5%) among RES, while current cigarette use was reported in 53 cases (26.6% overall; 34.0% in RRMS; 66.0% RES). Alcohol consumption was occasional in 116 patients (58.3% overall; 31.0% RRMS; 69.0% RES), absent or not within the past 12 months in 81 individuals (40.7% overall; 28.4% RRMS; 71.6% RES), and daily in two cases, both from the RES group.

### General medical history of the cohort

The median time from diagnosis to enrollment was 0.7 years (IQR 0.6–1.5) in the overall group (N = 199). This was similar in patients with RES (median 0.7 years, IQR 0.6–0.8) and longer in those with RRMS (median 2.8 years, IQR 1.6–3.9).

At baseline, 198 medical conditions were recorded across the cohort: 133 (67.2%) were concomitant diseases, 42 (21.2%) surgeries/procedures, and 23 (11.6%) past diseases; the distribution was similar in RRMS and RES (Table 2). A detailed tabulation by System Organ Class is provided in Supplementary Table S4.

**Table 2:** General medical history of patients with multiple sclerosis in the overall population and by groups

	Total (N = 199)	RRMS (N = 59)	RES (N = 140)
Time from diagnosis to enrollment, years, median (IQR)	0.7 (0.6–1.5)	2.8 (1.6—3.9)	0.7 (0.6—0.8)
Age at first symptoms, n (%)			
< 40 years	137 (69.2)	45 (32.8)	92 (67.2)
40–55 years	52 (26.3)	12 (23.1)	40 (76.9)
> 55 years	9 (4.5)	2 (22.2)	7 (77.8)
n (missing)*	198 (1)	59 (0)	139 (1)
Age at diagnosis, n (%)			
< 40 years	130 (65.3)	43 (33.1)	87 (66.9)
40–55 years	59 (29.6)	13 (22.0)	46 (78.0)
> 55 years	10 (5.0)	3 (30.0)	7 (70.0)
Any relevant past disease, surgery/procedure or concomitant disease at baseline, n (%)	83 (41.7)	30 (36.1)	53 (63.9)
Medical Condition type, n (%)			
Past disease	23 (11.6)	6 (8.6)	17 (13.3)
Surgery / Procedure	42 (21.2)	15 (21.4)	27 (21.1)
Concomitant disease	133 (67.2)	49 (70.0)	84 (65.6)
Total, n (%)	198 (100.0)	70 (100.0)	128 (100.0)
Familiar history of autoimmune diseases, n (%)	40 (20.1)	7 (11.9)	33 (23.6)
Familiar history of neoplasms, n (%)	41 (20.6)	15 (36.6)	26 (63.4)

Data are reported as numbers (%) or median (IQR)

*BMI* body mass index; *IQR* interquartile range (Q1–Q3); *N* number of patients with available data; *RRMS* relapsing–remitting multiple sclerosis; *RES*, rapidly evolving severe relapsing–remitting multiple sclerosis

Data refer to the eligible set (N = 199) unless otherwise specified

^*^Percentages of age at symptom onset were calculated based on the 198 patients with available information

A family history of autoimmune disease was present in 40 (20.1%) patients, including 7 (17.5%) with RRMS and 33 (82.5%) with RES. Among these, MS was the most frequently reported condition and was observed exclusively in the RES group (Table 2, Supplementary Table S5). A family history of neoplasms was reported in 41 (20.6%) participants, 15 (36.6%) with RRMS and 26 (63.4%) with RES (Table 2), with breast cancer (BC) being the most frequent tumor (3 of 15, 20.0% in RRMS; 6 of 26, 23.1% in RES; Supplementary Table S6).

### Initial symptoms and disability at diagnosis

Data on functional system involvement at disease onset were available for 187 patients. Among these, 116 patients (62.0%) presented a monofocal onset of MS while 71 (38.0%) had a multifocal onset. The most frequently affected functional systems were sensory (85 patients, 45.5%), pyramidal–lower limb (51, 27.3%), and visual (45, 24.1%) (Supplementary Figure S1A). At diagnosis, information on functional system involvement was available for 180 patients. Among these, 79 patients (43.9%) presented a monofocal involvement while 101 (56.1%) had a multifocal involvement. The affected functional systems were similar to the ones observed at time of disease onset (Supplementary Figure S1B). No relevant differences were observed between the RRMS and RES groups at either assessment.

At diagnosis, disability was generally mild: 147/196 patients (75.0%) had EDSS ≤ 2.5 (Supplementary Table S7). The most frequent EDSS categories were 2.0 in 23.0% of patients (45/196), 1.0 in 17.9% (35/196), and 2.5 in 13.8% (27/196). Scores ≥ 4.0 occurred in only 10/196 patients (5.1%). The distribution of EDSS scores by disease subtype, RRMS and RES, is shown in Supplementary Table S7.

### Clinical features at baseline

At baseline, the median EDSS score was 2.0 (IQR 1.0–3.0), and most patients scored between 1.0 and 3.5 (Table 1, Supplementary Table S8). The most frequent categories were 2.0 (42/199, 21.1%), 1.0 (39/199, 19.6%), and 3.5 (33/199, 16.6%). Relapses within the previous 12 months were reported in 105 of 199 (52.8%) patients, including 27 (25.7%) in the RRMS group and 78 (74.3%) in RES. Among patients with a recorded relapse, 65 (61.9%) had fully recovered without residual symptoms, 34 (32.4%) had residual symptoms, and 1 (1.0%) had an unresolved relapse. The mean number of relapses in the previous year was 0.7 overall (0.5 in RRMS, 0.8 in RES) (Supplementary Table S8).

The SDMT was performed in 150 of 199 (75.4%) patients. The mean corrected score for the SDMT was 48.1 ± 13.2, consistent across RRMS and RES (Supplementary Table S9). Other cognitive measures, including the Selective Reminding Test (SRT), Spatial Recall Test (SPART), Paced Auditory Serial Addition Test (PASAT), and Word List Generation (WLG), showed mean performance within the expected range for mildly affected MS patients (Supplementary Table S9).

### Prior disease-modifying therapies

Overall, 59 of 199 (29.6%) patients had received only one DMT before starting ocrelizumab, while 140 patients (70.4%) were treatment-naïve. Prior exposure was almost exclusively observed in the RRMS group, with only 1 RES patient, who had received a DMT for a single day before switching. Descriptive analyses of previous treatment duration and reasons for switch therefore refer to the 59 RRMS patients with evaluable data. The median time from the last DMT to ocrelizumab initiation was 1.4 months (IQR 0.7–2.0) (Table 3). Duration of the previous DMT was > 12 months in 40 (67.8%), 7–12 months in 13 (22.0%), and ≤ 6 months in 6 (10.2%) patients (Table 3). The most common reason for switching to ocrelizumab was MRI activity (new/enlarged T2-hyperintense or Gd + lesions; N = 28, 47.5%), followed by safety concerns (N = 23, 38.9%), MS relapse (N = 15, 25.0%), other reasons (N = 6, 10.2%), and increased EDSS score (N = 4, 6.7%). Reasons were not mutually exclusive, and multiple reasons could be recorded for the same patient (Table 3).

**Table 3:** Disease-modifying therapies (DMTs) prior to ocrelizumab treatment (N = 199)

	Total (N = 199)
N of patients with previous DMT	59 (30.1)
Time from previous DMT to the start of ocrelizumab (months), median (IQR)	1.4 (0.7—2.0)
Duration of previous DMT	
≤ 6 months	6 (10.2)
7—12 months	13 (22)
> 12 months	40 (67.8)
Reason for DMT switch to ocrelizumab*	
MRI activity (T1 Gd + lesions or new/enlarged T2 lesions)	28 (47.5)
Safety concerns	23 (38.9)
MS relapse	15 (25.0)
Other	6 (10.2)
Increased EDSS	4 (6.7)

Data are shown as n (%)

Sixty patients had received a previous DMT; descriptive analyses were based on the 59 RRMS patients with evaluable data, as the single RES patient had only one day of prior DMT exposure. Details on specific prior DMTs are reported in Supplementary Table S10

*DMT* disease-modifying therapy; *RRMS* relapsing–remitting multiple sclerosis; *RES* rapidly evolving severe relapsing–remitting multiple sclerosis; *MRI* magnetic resonance imaging; *Gd + *, gadolinium-enhancing; *EDSS* expanded disability status scale; *IQR* interquartile range

^*^ Reasons are not mutually exclusive; percentages use N = 59 as the denominator

The most frequently used treatment among the 59 patients with previous DMT were natalizumab (33.9%), dimethylfumarate (25.4%), cladribine (13.6%), and teriflunomide (10.2%), with injectable therapies used in a smaller proportion of patients (Supplementary Table S10). Switching from natalizumab was predominantly safety-driven (17/20, 85%), whereas dimethyl fumarate changes were mainly prompted by MRI activity (6/15, 40%) with additional multiple-reason reports (4/15, 26.7%). Among cladribine switches, multiple reasons were often reported (5/8, 62.5%), including MRI activity, which was cited in 3/8 cases (37.5%); injectable therapies likewise frequently had multiple reasons (4/6, 66.7%) (Supplementary Table S11).

At the individual-drug level (Supplementary Table S11), median exposure durations were 36.8 months for fingolimod, 30.6 (8.7–43.5) for injectable therapies (interferons/glatiramer), 20.4 (8.7–35.5) for natalizumab, 18.7 (11.9–36.7) for dimethyl fumarate, 18.2 (16.0–33.6) for teriflunomide, 15.5 (0.8–28.6) for ozanimod, and 12.4 (1.2–19.0) for cladribine.

### Magnetic Resonance Imaging features

MRI data at the time of diagnosis were available for 188/199 (94.5%) patients (RRMS N = 56; RES N = 132) (Table 4). Most patients underwent both brain and spinal cord MRI (146/188, 77.7%), while 40 (21.3%) had brain-only and 2 (1.1%) spinal cord–only MRIs. Gd was administered in 157/188 (83.5%) scans at diagnosis. The mean number of Gd + brain lesions was 1.6 ± 2.9 (1.7 ± 3.0 and 1.5 ± 2.8 for RRMS and RES, respectively). In the spinal cord, the mean number of Gd + lesions was 0.8 ± 1.6 in all groups. Among 157 patients with available data, 98 (62.4%) presented ≥ 9 T2-hyperintense brain lesions, 48 (30.6%) had 3–8 lesions, and only 1 (0.6%) had no lesions. A similar distribution was observed in RRMS and RES. In the spinal cord, evaluable T2-hyperintense lesion data were available for 129/199 patients (64.8% of the overall cohort): 72 (55.8%) had ≥ 3 lesions, while 14 (10.8%) had no cord lesions. The mean interval between diagnostic and baseline MRI was 1.0 ± 1.3 years (N = 177) (Supplementary Table S12).

**Table 4:** MRI findings at diagnosis in the overall population and in RRMS and RES groups

	Total (N = 199)	RRMS (N = 59)	RES (N = 140)
Scanned region, n (%)			
N (missing)	188 (11)	56 (3)	132 (8)
Brain only, n (%)	40 (21.3)	15 (37.5)	25 (62.5)
Spinal cord only, n (%)	2 (1.1)	1 (50.0)	1 (50.0)
Brain and spinal cord, n (%)	146 (77.7%)	40 (27.4)	106 (72.6)
Gadolinium-based contrast administered,			
N (missing)	188(11)	56 (3)	132 (8)
n (%)	157 (83.5)	48 (30.6)	109 (69.4)
Gadolinium-enhancing lesion count in the brain			
N (missing)	155(44)	47 (12)	108 (32)
mean ± SD	1.6 ± 2.9	1.7 ± 3.0	1.5 ± 2.8
Gadolinium-enhancing lesion count in the spinal cord			
N (missing)	126(73)	36 (23)	90 (50)
mean ± SD	0.8 ± 1.6	0.8 ± 1.6	0.8 ± 1.6
T2-hyperintense lesion count in the brain			
N (missing)	157 (42)	42 (17)	115 (25)
0 T2 lesions, n (%)	1 (0.6)	0 (0.0)	1 (100.0)
1—2 T2 lesions, n (%)	10 (6.4)	3(30.0)	7 (70.0)
3—8 T2 lesions, n (%)	48 (30.6)	14(29.2)	34 (70.8)
≥ 9 T2 lesions, n (%)	98 (62.4)	25(25.5)	73 (74.5)
T2-hyperintense lesion count in the spinal cord			
N (missing)	129 (70)	35 (24)	94 (46)
0 T2 lesions, n (%)	14 (10.8)	3 (21.4)	11 (78.6)
1—2 T2 lesions, n (%)	43 (33.4)	17 (39.5)	26 (60.5)
≥ 3T2 lesions, n (%)	72 (55.8)	15 (20.8)	57 (79.2)

Percentages for RRMS and RES are calculated using, as the denominator, the total number of patients identified within each specific category

Values are expressed as n (%) and mean (SD)

“Valid N” indicates the number of patients with available data for each variable

*RRMS* relapsing–remitting multiple sclerosis; *RES* rapidly evolving severe relapsing–remitting multiple sclerosis; *SD* standard deviation

Baseline MRI was available for local reading in all 199 patients. Brain MRI was performed in 100% of cases, and spinal cord imaging in 138 (69.3%). Gd was administered in 166 (83.4%) patients. At baseline, the mean number of Gd + lesions was 0.8 ± 2.2 in the brain (N = 164) and 0.4 ± 1.3 in the spinal cord (N = 117) (Supplementary Table S12). Among 193 patients with available brain MRI data, ≥ 9 lesions were present in 116 (60.1%), and 65 (33.7%) had 3–8 lesions. New or enlarged T2-hyperintense lesions compared with the last available scan prior to baseline were identified in 33 patients (mean 3.0 ± 4.2). In the spinal cord, among 138 patients with evaluable data, ≥ 3 T2-hyperintense lesions were observed in 81 (40.7%), and new/enlarged lesions were reported in 13 cases (Supplementary Table S12).

Centralized analysis measures of baseline MRI are reported in Supplementary Table S12. T2-hyperintense LV could be measured in 173 (87%) patients, mean T2-hyperintense LV was 4.64 ± 5.53 ml. T1-hypointense LV could be measured in 107 (54%) patients, mean T1-hypointense LV was 1.14 ± 2.05 ml. Centralized Gd + lesion count was performed in 152 (76%) patients, mean Gd + lesion count was 0.5 ± 2.0.

## Discussion

This study presents the first interim analysis of the MUSPO cohort, providing a comprehensive description of the baseline demographic, clinical, and MRI characteristics of the enrolled Italian RRMS and RES groups. The low proportion of ineligible patients (4%) and broad geographic participation confirm the feasibility of enrolling a representative national sample under non-interventional conditions. Furthermore, 36% of patients joined the immunological substudy, enabling future analyses of immune phenotyping and immune dynamics and of treatment response heterogeneity, a feature rarely available in observational settings. These findings offer valuable insights into most recent treatment paradigms and patient profiles in Italy, serving as a reference framework for interpreting future longitudinal effectiveness results and complementing evidence from pivotal trials.

Compared with other European and Italian real-world cohorts, such as CONFIDENCE and MUSICALE [28, 29], and the retrospective study by Zaccone et al. [30], the MUSPO population is slightly younger and less disabled, according to the study’s enrollment criteria. The median age of our cohort at enrollment was below 40 years, and the majority were female, consistent with the known epidemiology of MS [31]. In line with this female predominance, BC was the most frequently reported tumor in the familiar history of our cohort (20—23%), consistent with global cancer statistics indicating that BC accounts for approximately 24% of all cancers in women worldwide [32]. The predominance of the RES group (70%) reflects the treatment practice in Italy at the time of enrollment, when neurologists generally selected ocrelizumab for patients with highly active or aggressive disease courses.

The median time from diagnosis to study enrollment was approximately 0.7 years; this short interval is aligned with current/recent treatment guidelines recommending the early use of high-efficacy agents to prevent irreversible damage [15, 33]. Baseline disability was mild (median EDSS = 2.0), with nearly three-quarters of patients scoring ≤ 2.5. This low baseline disability is consistent with an early treatment initiation strategy and is comparable to the patient profiles observed at enrollment in the pivotal OPERA trials (mean EDSS = 2.8) [13, 15]**.** It also provides an ideal condition to evaluate NEDA-plus outcomes, since early treatment with ocrelizumab in low-disability patients has been associated with better functional prognosis [15, 34]**.**

The analysis of prior DMT exposure in the MUSPO cohort showed that 29.6% (N = 59) of patients had received one prior DMT, while 70.4% (N = 140) were treatment-naïve. Among patients with prior DMT exposure, natalizumab and dimethyl fumarate were the most frequent prior therapies, a pattern consistent with recent Italian registry data [35]. The most common reason for switching was MRI activity (46.7% of switchers) and safety concerns (38.3%), comparable to prior observational findings [28] and current consensus recommendations that encourage rapid escalation upon evidence of subclinical inflammatory activity [27]. The median time from DMT discontinuation to ocrelizumab initiation was only 1.4 months (IQR 0.7–2.0), suggesting efficient treatment transition and proper washout intervals, an important clinical strategy for minimizing the risk of disease reactivation following the discontinuation of potent agents like natalizumab [36].These results confirm that ocrelizumab is often chosen as a second-line therapy when breakthrough activity occurs or when safety concerns limit continued use of other DMTs, according to the enrollment criteria.

MRI findings provide further insight into the inflammatory profile of the cohort at the time of ocrelizumab initiation. MRI data at diagnosis and baseline were available for almost all patients, with combined brain and spinal cord imaging performed in approximately two-thirds of cases at both time points. Gd contrast was administered in 157/188 scans (83.5%). The overall lesion burden, including a mean of 0.8 Gd + brain lesions at baseline and a high frequency of T2-hyperintense lesions (98/157, 62.4% with ≥ 9 lesions), indicated moderate inflammatory activity at the time of ocrelizumab initiation, despite the short median disease duration. This finding is consistent with the current treatment guidelines advocating the early use of high-efficacy therapy to rapidly suppress any measurable inflammatory activity [15, 33]. The overall baseline inflammatory burden appears lower than that reported in other Italian real-world cohorts on ocrelizumab, suggesting that MUSPO may capture a slightly earlier stage of the disease course [35].

The systematic prospective collection of advanced cognitive and PROs is a major strength of the MUSPO study, addressing recognized limitations in traditional real-world cohorts. Incorporation of cognitive testing into a large observational cohort is a notable methodological feature, as cognitive decline is often under-recognized in routine clinical practice and yet contributes significantly to disability and quality of life [37]. The annual collection of validated PROs (including MSIS-29 [23], MFIS [38], TSQM [25], and MS-COI [39]) will provide information on patient-perceived impact, fatigue, and satisfaction, completing the multidimensional characterization of treatment outcomes beyond traditional clinical and MRI measures.

### Study limitations

There are some limitations of the present study that need to be mentioned. The descriptive nature of this interim analysis and the lack of a comparator arm. Although the inclusion criteria were broad and encompassed two predefined groups, the predominance of early or highly active disease may influence the overall results that will be observed in the enrolled population. The proportion of the overall national population of potentially eligible patients included in the study was not assessed, which may limit the evaluation of the representativeness of the cohort. In this regard, future studies may benefit from broader site participation and more comprehensive patient inclusion to enhance representativeness. Detailed information on ethnicity, socioeconomic status, and educational level, as well as data on patients who declined participation or were not invited, were not collected, which may limit the assessment of equity of access and potential barriers to inclusion. In addition, enrollment approximately six months after treatment initiation may introduce a potential selection bias, as patients who discontinued early or experienced severe adverse events may not have been captured. Furthermore, the lack of pre-treatment baseline biological samples limits the interpretation of immunological findings. Finally, the present data focus solely on describing the baseline characteristics of the study population, who at this stage have had only a relatively short exposure period.

## Conclusion

This interim analysis provides a detailed demographic and clinical profile of Italian patients with RRMS treated with ocrelizumab, depicting a predominantly young, mildly disabled, and early-treated cohort according to inclusion criteria. These characteristics are of particular relevance in an observational setting related to ocrelizumab and their role will be evaluated in the framework of future effectiveness results. The study primary objective will determine the proportion of patients achieving NEDA-plus and evaluate MRI-based brain and spinal atrophy, cognitive outcomes, and PROs after a 4-year follow up. The MUSPO cohort will provide robust real-world evidence on 4-years effectiveness and safety of early treatment with ocrelizumab in Italian clinical practice.

## Supplementary Information

Below is the link to the electronic supplementary material.

Supplementary file1 (DOCX 35 KB)

Supplementary file2 (DOCX 57 KB)

## Data Availability

The data that support the findings of this study are available on reasonable request from the corresponding author. The data are not publicly available due to privacy or ethical restrictions. The Statistical Analysis Plan (SAP) and additional methodological details can also be made available upon reasonable request through the corresponding author.
