# Supplementary material for: Ocrelizumab in early relapsing–remitting multiple sclerosis: first interim analysis of the MUSPO Italian prospective cohort
Source: J Neurol. 2026 May 4;273(5):302. doi: 10.1007/s00415-026-13830-0 (PMC13171665; doi:10.1007/s00415-026-13830-0)
Supplement: Supplementary file 1 — Supplementary file1 (DOCX 35 KB) [file 415_2026_13830_MOESM1_ESM.docx]

**Supplementary figure S1.** Functional systems involved at the time of the first symptoms (A) and at diagnosis (B) in the overall population and by groups. Bars show, for each functional system (sensory, pyramidal–lower limb, pyramidal–upper limb, visual, brainstem, cerebellar, bowel and bladder, ambulation, cerebral, cognition), the percentage of patients with involvement. Percentages were calculated among patients with available data (N = 187*). Abbreviations: RRMS, relapsing–remitting multiple sclerosis; RES, rapidly evolving severe multiple sclerosis.*

A)

B)
