# Supplementary material for: Ocrelizumab in early relapsing–remitting multiple sclerosis: first interim analysis of the MUSPO Italian prospective cohort
Source: J Neurol. 2026 May 4;273(5):302. doi: 10.1007/s00415-026-13830-0 (PMC13171665; doi:10.1007/s00415-026-13830-0)
Supplement: Supplementary file 2 — Supplementary file2 (DOCX 57 KB) [file 415_2026_13830_MOESM2_ESM.docx]

**Supplementary table S1**. Eligibility criteria for enrolled patients.

| **INCLUSION CRITERIA** | |
| --- | --- |
| **Criterion** | **Definition** |
| Inclusion criteria A | Patient has signed the informed consent |
| Inclusion criteria B | Patient has ≥ 18 years of age |
| Inclusion criteria C | Patient has an EDSS score ≤ 3.5 |
| Inclusion criteria D* | - Original Protocol v1.0 (20/03/2023): Patient has a recent (≤ 2 years) definite diagnosis of RR according to McDonald 2017 and for whom a decision to start ocrelizumab has been taken in routine practice. - Emended Protocol v2.0 (03/05/2024): Patient has a recent definite diagnosis of RR according to the current version of McDonald criteria, defined as ≤ 5 years for patients in Criterion #1 and ≤ 2 years for patients in Criterion #2, and for whom a decision to start ocrelizumab has been taken in routine practice. |
| Inclusion criteria E | Patient is able and willing to complete the PROs questionnaires |
| Inclusion criteria F | Patient has received the first infusion of ocrelizumab around 6 months before study entry, using it per the current SmPC and local label according to the following criteria:   - Criterion #1: RR with inadequate response to exactly one prior DMT after a complete course (e.g., ≥6 months–1 year), with ≥1 relapse in the previous year while on therapy and ≥1 new Gd+ lesion or a new/enlarged T2 lesion on a recent MRI. Patients who discontinued the only prior DMT for safety/tolerability per local labeling are also eligible. - Criterion #2: Rapidly evolving severe (RES) RR defined as ≥2 disabling relapses in one year and ≥1 new Gd+ lesion or significantly increased T2 lesion load on brain MRI. |
| Inclusion criteria G | Patient who has not yet received the second dose (third infusion) of ocrelizumab. |
| **EXCLUSION CRITERIA** | |
| **Criterion** | **Definition** |
| Exclusion criteria A | Patient not receiving treatment for RR with ocrelizumab according to standard of care and in line with the current SmPC/local labeling. |
| Exclusion criteria B | Patients previously treated with any anti-CD20 antibodies before entering the study. |

* Inclusion criterion D differed between protocol versions. In v1.0, eligibility required a diagnosis within ≤2 years. In v2.0, the diagnostic time window was expanded to ≤5 years for patients meeting Criterion #1 (RRMS with inadequate response to exactly one prior DMT) and remained ≤2 years for those meeting Criterion #2 (rapidly evolving severe RRMS).

**Supplementary Table S2.** Study set-up, screening outcomes, and protocol versions.

| Metric | Value |
| --- | --- |
| Sites, n | 31 |
| First / last date of informed consent (eligible set) | 29-Sep-2023 / 19-May-2025 |
| Inclusion criteria (enrolled/met), n (%) |  |
| *criteria A–C, E* | 208 (100) |
| *criterion D* |  |
| *v1.0* | 40 (97.6) |
| *v2.0* | 162 (98.2) |
| *criterion F* | 188 (90.4) |
| *criterion G* | 206 (99.0) |
| Exclusion criteria (enrolled/present), n (%) |  |
| *criterion A* | 0 (0) |
| *criterion B* | 1 (0.5) |
| Enrolled by protocol version, n (%) |  |
| *v1.0* | 41 (19.7) |
| *v2.0* | 167 (80.3) |
| Eligible by protocol version, n (%) |  |
| *v1.0* | 39 (19.6) |
| *v2.0* | 160 (80.4) |

Data are reported as number (%).

Percentages for inclusion/exclusion criteria are calculated on the enrolled set (N=208). Criterion D is reported separately by protocol version because its definition differed between v1.0 and v2.0. Protocol version v1.0 (20 March 2023) required a diagnosis within ≤2 years. Protocol version v2.0 (03 May 2024) expanded the allowable timeframe to ≤5 years for patients meeting Criterion #1 (*relapsing-remitting multiple sclerosis,* RRMS, with inadequate response to exactly one prior DMT) and ≤2 years for those meeting Criterion #2 (rapidly evolving severe RRMS, RES).

*Abbreviations. v1.0/v2.0: protocol version 1.0 (original)/2.0 (emended); eligible: patients meeting all eligibility criteria and included in the analysis set; enrolled/met: criterion fulfilled/satisfied among enrolled patients; enrolled/present: exclusion condition present (patient not eligible) among enrolled patients.*

**Supplementary Table S3.** Enrolled patients (N=208) per site (N=31) and protocol version.

| **site** | **Total** | **v.1** | **v.2** |
| --- | --- | --- | --- |
| 3 | 22 (10.6) | 9 (22.0) | 13 (7.8) |
| 25 | 22 (10.6) | 4 (9.8) | 18 (10.8) |
| 2 | 16 (7.7) | 1 (2.4) | 15 (9.0) |
| 29 | 12 (5.8) | 0 (0.0) | 12 (7.2) |
| 7 | 11 (5.3) | 7 (17.1) | 4 (2.4) |
| 33 | 8 (3.8) | 0 (0.0) | 8 (4.8) |
| 28 | 8 (3.8) | 0 (0.0) | 8 (4.8) |
| 24 | 8 (3.8) | 2 (4.9) | 6 (3.6) |
| 5 | 8 (3.8) | 3 (7.3) | 5 (3.0) |
| 27 | 7 (3.4) | 0 (0.0) | 7 (4.2) |
| 18 | 7 (3.4) | 0 (0.0) | 7 (4.2) |
| 9 | 7 (3.4) | 2 (4.9) | 5 (3.0) |
| 22 | 6 (2.9) | 3 (7.3) | 3 (1.8) |
| 19 | 6 (2.9) | 3 (7.3) | 3 (1.8) |
| 13 | 6 (2.9) | 3 (7.3) | 3 (1.8) |
| 8 | 6 (2.9) | 0 (0.0) | 6 (3.6) |
| 4 | 6 (2.9) | 1 (2.4) | 5 (3.0) |
| 11 | 5 (2.4) | 0 (0.0) | 5 (3.0) |
| 10 | 5 (2.4) | 0 (0.0) | 5 (3.0) |
| 23 | 4 (1.9) | 0 (0.0) | 4 (2.4) |
| 20 | 4 (1.9) | 0 (0.0) | 4 (2.4) |
| 14 | 4 (1.9) | 0 (0.0) | 4 (2.4) |
| 21 | 3 (1.4) | 0 (0.0) | 3 (1.8) |
| 15 | 3 (1.4) | 2 (4.9) | 1 (0.6) |
| 1 | 3 (1.4) | 0 (0.0) | 3 (1.8) |
| 31 | 2 (1.0) | 0 (0.0) | 2 (1.2) |
| 30 | 2 (1.0) | 0 (0.0) | 2 (1.2) |
| 26 | 2 (1.0) | 0 (0.0) | 2 (1.2) |
| 17 | 2 (1.0) | 0 (0.0) | 2 (1.2) |
| 12 | 2 (1.0) | 1 (2.4) | 1 (0.6) |
| 32 | 1 (0.5) | 0 (0.0) | 1 (0.6) |

Data are reported as numbers (%).

**Supplementary Table S4.** Medical conditions reported at baseline in the overall population, summarized by System Organ Class (SOC).

Percentages are calculated on the total number of reported medical conditions (N = 198).

| **Medical Condition by SOC, n(%)** |  |
| --- | --- |
| Surgical and medical procedures | 40 (20.2) |
| Metabolism and nutrition disorders | 27 (13.6) |
| Psychiatric disorders | 16 (8.1) |
| Endocrine disorders | 15 (7.6) |
| Musculoskeletal and connective tissue disorders | 14 (7.1) |
| Vascular disorders | 12 (6.1) |
| Nervous system disorders | 9 (4.5) |
| Gastrointestinal disorders | 9 (4.5) |
| Congenital, familial and genetic disorders | 8 (4.0) |
| Skin and subcutaneous tissue disorders | 6 (3.0) |
| Neoplasms benign, malignant and unspecified (incl cysts and polyps) | 6 (3.0) |
| Infections and infestations | 6 (3.0) |
| Reproductive system and breast disorders | 5 (2.5) |
| Cardiac disorders | 5 (2.5) |
| Investigations | 4 (2.0) |
| Respiratory, thoracic and mediastinal disorders | 3 (1.5) |
| Renal and urinary disorders | 3 (1.5) |
| Injury, poisoning and procedural complications | 3 (1.5) |
| Hepatobiliary disorders | 3 (1.5) |
| Blood and lymphatic system disorders | 2 (1.0) |
| Immune system disorders | 1 (0.5) |
| Eye disorders | 1 (0.5) |

Data are reported as numbers (%).

**Supplementary table S5**. Type of family history for autoimmune diseases in the RRMS and RES groups.

| **RRMS**  **(N=59)** | | **RES**  **(N=140)** | |
| --- | --- | --- | --- |
| *Patient ID* | *Autoimmune Disorder* | *Patient ID* | *Autoimmune Disorder* |
| RRMS-01 | Diabetes Mellitus | RES-01 | Autoimmune Thyroiditis |
| RRMS-02 | Immune-mediated pathology of central nervous system | RES-02 | Cancer |
| RRMS-03 | Dysthyroidism (other), Diabetes Mellitus Type I | RES-03 | Diabetes Mellitus |
| RRMS-04 | Hashimoto's Thyroiditis | RES-04 | Diabetes Mellitus, Hyperthyroidism |
| RRMS-05 | Hashimoto's Thyroiditis, Multiple Sclerosis | RES-05 | Hashimoto's Thyroiditis |
| RRMS-06 | Rheumatoid Arthritis | RES-06 | Hashimoto's Thyroiditis, Multiple Sclerosis |
| RRMS-07 | Rheumatoid Arthritis | RES-07 | Immune Thrombocytopenia |
|  |  | RES-08 | Miastenia Gravis |
|  |  | RES-09 | Ankylosing Spondylitis, Crohn's Disease |
|  |  | RES-10 | Multiple Sclerosis |
|  |  | RES-11 | Multiple Sclerosis |
|  |  | RES-12 | Multiple Sclerosis |
|  |  | RES-13 | Multiple Sclerosis |
|  |  | RES-14 | Multiple Sclerosis |
|  |  | RES-15 | Multiple Sclerosis |
|  |  | RES-16 | Multiple Sclerosis |
|  |  | RES-17 | Multiple Sclerosis |
|  |  | RES-18 | Multiple Sclerosis |
|  |  | RES-19 | Multiple Sclerosis |
|  |  | RES-20 | Multiple Sclerosis |
|  |  | RES-21 | Multiple Sclerosis |
|  |  | RES-22 | Multiple Sclerosis |
|  |  | RES-23 | Multiple Sclerosis |
|  |  | RES-24 | Multiple Sclerosis |
|  |  | RES-25 | Multiple Sclerosis |
|  |  | RES-26 | Multiple Sclerosis  Familiar Mediterranean Fever |
|  |  | RES-27 | Psoriasis |
|  |  | RES-28 | Psoriasis |
|  |  | RES-29 | Rheumatoid Arthritis |
|  |  | RES-30 | Rheumatoid Arthritis |
|  |  | RES-31 | Rheumatoid Arthritis, Psoriasis, Scleroderma |
|  |  | RES-32 | RRMS |
|  |  | RES-33 | Walking Disorders And Abstemiousness |

*Abbreviations: RRMS, relapsing-remitting multiple sclerosis; RES, rapidly evolving severe relapsing-remitting multiple sclerosis.*

**Supplementary table S6.** Type of family history of neoplasms in the RRMS and RES groups.

| **RRMS**  **(N=59)** | | **RES**  **(N=140)** | |
| --- | --- | --- | --- |
| *Patient ID* |  | *Patient ID* |  |
| RRMS-01 | Brain tumor (glioblastoma) | RES-34 | Bladder neoplasm |
| RRMS-08 | Breast cancer | RES-29 | Brain tumor |
| RRMS-06 | Breast cancer | RES-35 | Breast cancer |
| RRMS-09 | Colorectal cancer | RES-07 | Breast cancer |
| RRMS-10 | Gastrointestinal neoplasm | RES-36 | Breast cancer |
| RRMS-03 | Hodgkin's lymphoma; breast cancer | RES-37 | Breast cancer; colon carcinoma; lung carcinoma; melanoma |
| RRMS-11 | Liver carcinoma | RES-31 | Breast cancer; kidney cancer |
| RRMS-12 | Lung cancer | RES-38 | Breast cancer |
| RRMS-13 | Lung cancer | RES-39 | Colon carcinoma; oesophageal carcinoma |
| RRMS-14 | Lung cancer; colon cancer | RES-40 | Colorectal cancer |
| RRMS-15 | Melanoma | RES-41 | Cutaneous lymphoma |
| RRMS-16 | Multiple myeloma | RES-42 | Data unknown |
| RRMS-17 | Ovarian cancer | RES-43 | Gastric and laryngeal carcinoma |
| RRMS-18 | Pancreatic cancer | RES-44 | Gastric cancer |
| RRMS-19 | Unknown origin | RES-02 | Gastric cancer |
|  |  | RES-45 | Intestinal carcinoma |
|  |  | RES-46 | Lung cancer |
|  |  | RES-47 | Lung carcinoma |
|  |  | RES-48 | Mesothelioma |
|  |  | RES-09 | Mesothelioma |
|  |  | RES-49 | Multiple myeloma |
|  |  | RES-50 | Chronic myeloid leukemia |
|  |  | RES-51 | Oral cavity neoplasm |
|  |  | RES-13 | Stomach cancer |
|  |  | RES-52 | Thyroid cancer |
|  |  | RES-53 | Visceral cancer |

*Abbreviations: RRMS, relapsing-remitting multiple sclerosis; RES, rapidly evolving severe relapsing-remitting multiple sclerosis.*

**Supplementary table S7.** Expanded Disability Status Scale (EDSS) at diagnosis in the overall population (N = 199). Percentages are calculated on the 196 patients with available EDSS scores at diagnosis (3 missing values). Data are presented for the overall population and by groups: RRMS (N = 58) and RES (N = 138).

| **EDSS score** | **Total** | **RRMS** | **RES** |
| --- | --- | --- | --- |
| 0 | 16 (8.0) | 7 (43.8) | 9 (56.2) |
| 1 | 35 (17.6) | 16 (45.7) | 19 (54.3) |
| 1.5 | 24 (12.1) | 6 (25.0) | 18 (75.0) |
| 2 | 45 (22.6) | 12 (26.7) | 33 (73.3) |
| 2.5 | 27 (13.6) | 8 (29.6) | 19 (70.4) |
| 3 | 20 (10.1) | 3 (15.0) | 17 (85.0) |
| 3.5 | 19 (9.5) | 1 (5.3) | 18 (94.7) |
| 4 | 7 (3.5) | 3 (42.9) | 4 (57.1) |
| 4.5 | 2 (1.0) | 2 (100.0) | 0 (0.0) |
| 5 | 0 (0.0) | 0 (–) | 0 (–) |
| 5.5 | 1 (0.5) | 0 (0.0) | 1 (100.0) |
| 6-10 | 0 (0.0) | 0 (–) | 0 (–) |

Values are expressed as n (% of EDSS category total).

– indicates that no patients were present in that category, and percentage calculation was therefore not applicable.

*Abbreviations: EDSS, Expanded Disability Status Scale; RRMS, relapsing-remitting multiple sclerosis; RES, rapidly evolving severe relapsing-remitting multiple sclerosis.*

**Supplementary table S8.** Multiple sclerosis status at baseline in the overall population and by groups (RRMS, RES). Percentages of RRMS and RES for each variable are calculated based on the denominator with respect to the corresponding total population.

|  | **Total**  **(N=199)** | **RRMS**  **(N=59)** | **RES**  **(N=140)** |
| --- | --- | --- | --- |
| *EDSS score at baseline, n (%)* |  |  |  |
| 0 | 15 (7.5) | 5 (33.3) | 10 (66.7) |
| 1 | 39 (19.6) | 13 (33.3) | 26 (66.7) |
| 1.5 | 31 (15.6) | 9 (29.0) | 22 (71.0) |
| 2 | 42 (21.1) | 12 (28.6) | 30 (71.4) |
| 2.5 | 21 (10.6) | 9 (42.9) | 12 (57.1) |
| 3 | 18 (9.0) | 5 (27.8) | 13 (72.2) |
| 3.5 | 33 (16.6) | 6 (18.2) | 27 (81.8) |
| *Any relapse in past 12 months, n (%)* |  |  |  |
| Yes | 105 (52.8) | 27 (25.7) | 78 (74.3) |
| Unknown | 3 (1.5) | 1 (33.3) | 2 (66.7) |
| *Outcome of most recent relapse, n (%)** |  |  |  |
| Resolved without residual symptoms | 65 (61.9) | — | — |
| Resolved with residual symptoms | 34 (32.4) | — | — |
| Not resolved | 1 (1.0) | — | — |
| Unknown | 5 (4.8) | — | — |
| Mean number of relapses in past 12 months | 0.7 | 0.5 | 0.8 |

Data are expressed as number of patients (% of category total) or mean.

*Notes: Percentages of the status of most recent relapse are calculated on the 105 patients that reported at least one relapse in the past 12 months.*

*Abbreviations: RRMS, relapsing-remitting multiple sclerosis; RES. rapidly evolving severe relapsing-remitting multiple sclerosis; EDSS: Expanded Disability Status Scale.*

**Supplementary table S9**. Baseline cognitive function evaluation in the overall population and by groups (RRMS, RES). For each test, the table reports: performed, n (%): number (percentage) of patients who completed the test; raw score, mean (SD): unadjusted test score as obtained directly from test administration; corrected score, mean (SD): demographically adjusted standardized score based on normative data from Amato et al.[1] (adjusted for age, sex, and education). Percentages of RRMS and RES for each variable are calculated based on the denominator with respect to the corresponding total population. Missing data were not imputed. Higher scores indicate better performance.

| **Cognitive test** | **Metric** | **Total (N=199)** | **RRMS**  **(N=59)** | **RES**  **(N=140)** |
| --- | --- | --- | --- | --- |
| **SDMT** | Performed, n (%) | 150 (75.4) | 41 (69.5) | 109 (77.9) |
|  | Raw score, mean (SD) | 49.9 (11.8) | 50.7 (11.9) | 49.5 (11.8) |
|  | Corrected score, mean (SD) | 48.1 (13.2) | 51.4 (10.0) | 47.0 (14.0) |
| **SRT-LTS** | Performed, n (%) | 28 (14.1) | 5 (8.5) | 23 (16.4) |
|  | Raw score, mean (SD) | 37.1 (13.6) | 36.4 (13.8) | 37.3 (13.8) |
|  | Corrected score, mean (SD) | 37.1 (15.6) | 49 | 32.9 (15.7) |
| **SRT-CLTR** | Performed, n (%) | 28 (14.1) | 5 (8.5) | 23 (16.4) |
|  | Raw score, mean (SD) | 30.7 (15.1) | 24.0 (18.3) | 32.1 (14.4) |
|  | Corrected score, mean (SD) | 27.7 (14.6) | 42.5 | 26.2 (14.4) |
| **SRT-D** | Performed, n (%) | 28 (14.1) | 5 (8.5) | 23 (16.4) |
|  | Raw score, mean (SD) | 7.7 (2.5) | 8.4 (2.7) | 7.6 (2.5) |
|  | Corrected score, mean (SD) | 7.9 (1.9) | 11.3 | 7.6 (1.6) |
| **SPART** | Performed, n (%) | 28 (14.1) | 5 (8.5) | 23 (16.4) |
|  | Raw score, mean (SD) | 18.5 (6.4) | 20.4 (1.8) | 18.0 (6.9) |
|  | Corrected score, mean (SD) | 14.9 (5.1) | 19.7 | 14.4 (6.9) |
| **SPART-D** | Performed, n (%) | 28 (14.1) | 5 (8.5) | 23 (16.4) |
|  | Raw score, mean (SD) | 6.0 (2.5) | 6.2 (2.0) | 6.0 (2.6) |
|  | Corrected score, mean (SD) | 5.2 (3.0) | 4.5 | 5.2 (3.2) |
| **PASAT-3** | Performed, n (%) | 12 (6.0) | 1 (1.7) | 11 (7.9) |
|  | Raw score, mean (SD) | 35.9 (15.4) | 34.9 | 35.5 (16.1) |
|  | Corrected score, mean (SD) | 31.1 (16.4) | 30.7 | 30.7 (7.2) |
| **PASAT-2** | Performed, n (%) | 9 (4.5) | 1 (1.7) | 8 (5.7) |
|  | Raw score, mean (SD) | 23.9 (9.2) | 23.4 (9.7) | 23.4 (9.7) |
|  | Corrected score, mean (SD) | 22.1 (13.0) | 21.8 (14.1) | 21.8 (14.1) |
| **WLG** | Performed, n (%) | 27 (13.6) | 5 (8.5) | 22 (15.7) |
|  | Raw score, mean (SD) | 27.7 (8.9) | 27.0 (7.7) | 27.9 (9.3) |
|  | Corrected score, mean (SD) | 23.6 (12.9) | 22.1 | 23.7 (12.9) |

Data are expressed as mean (SD) and number of patients (%). SD was not reported when not computable because only one observation was available.

*Abbreviations* *SDMT, Symbol Digit Modalities Test (processing speed/attention); SRT, Selective Reminding Test; SRT-LTS, SRT Long-Term Storage (verbal learning/retention); SRT-CLTR, SRT Consistent Long-Term Retrieval (stable long-term retrieval); SRT-D, SRT Delayed Recall (delayed verbal recall); SPART, Spatial Recall Test (immediate visuospatial memory); SPART-D, SPART Delayed Recall (delayed visuospatial memory); PASAT-3, Paced Auditory Serial Addition Test, 3-second version (working memory/attention); PASAT-2, Paced Auditory Serial Addition Test, 2-second version; WLG, Word List Generation (verbal fluency/executive function).*

1. Amato MP, Portaccio E, Goretti B, et al (2006) The Rao’s Brief Repeatable Battery and Stroop Test: Normative Values with Age, Education and Gender Corrections in an Italian Population. Mult Scler 12:787–793. https://doi.org/10.1177/1352458506070933.

**Supplementary table S10.** Previous disease-modifying therapies (DMTs) among RRMS patients with prior DMT exposure (N=59).

|  | **Total** |
| --- | --- |
| Natalizumab | 20 (33.9) |
| Dimethyl fumarate | 15 (25.4) |
| Cladribine | 8 (13.6) |
| Teriflunomide | 6 (10.2) |
| Glatiramer acetate | 3 (5.1) |
| Ozanimod | 3 (5.1) |
| Interferon beta-1a | 2 (3.4) |
| Fingolimod | 1 (1.7) |
| Peginterferon beta-1a | 1 (1.7) |
|  |  |

Data are shown as n (%); percentages may not add up to 100% due to rounding.

*Abbreviations: RRMS, relapsing-remitting multiple sclerosis.*

**Supplementary table S11.** Duration of prior disease modifying therapies (DMTs) and reasons for switching to ocrelizumab, reported per individual DMT. Multiple reasons could be recorded for the same patient.

|  | **Dimethyl-fumarate** | **Fingolimod** | **Injectable therapy** | **Natalizumab** | **Ozanimod** | **Teriflunomide** | **Cladribine** |
| --- | --- | --- | --- | --- | --- | --- | --- |
| Valid N | N=15 | N=1 | N=6 | N=20 | N=3 | N=6 | N=8 |
| *Duration of previous DMT (months), Median (IQR)* | 18.7  (11.9-36.7) | 36.8  (NA) | 30.6  (8.7-43.5) | 20.4  (8.7-35.5) | 15.5  (0.8-28.6) | 18.2  (16.0-33.6) | 12.4  (1.2-19.0) |
|  |  |  |  |  |  |  |  |
| Reasons for DMT switch to ocrelizumab, n(%) |  |  |  |  |  |  |  |
| MS relapse | 3(20) | 0 | 0 | 0 | 0 | 0 | 0 |
| MRI activity (T1 Gd+ active lesions or new/enlarged T2 lesions) | 6(40) | 1(100) | 1(16.7) | 1(5) | 1(33.3) | 2(33.3) | 3(37.5) |
| Increased EDSS | 1(6.7) | 0 | 0 | 0 | 1(33.3) | 0 | 0 |
| Safety concerns | 1(6.7) | 0 | 0 | 17(85) | 1(33.3) | 0 | 0 |
| Other | 0 | 0 | 1(16.7) | 0 | 0 | 2(33.3) | 0 |
| Multiple reasons reported | 4(26.7) | 0 | 4(66.6) | 2(10) | 0 | 2(33.3) | 5(62.5) |

Data are presented as median (IQR) and n (%).

**Supplementary Table S12.** MRI findings at baseline in the overall population. Lesion counts are derived from site-reported baseline MRIs; centralized reading (CRC) data were available for a subset of cases. Percentages of RRMS and RES for each variable are calculated using, as the denominator, the total number of patients identified within each specific category.

|  | |  | Total  (N=199) | RRMS  (N=59) | RES  (N=140) |
| --- | --- | --- | --- | --- | --- |
| Time difference between diagnostic MRI and baseline MRI (years) | | N of patients (missing) | 177 (22) | 55 (4) | 122 (18) |
|  |  | Mean (SD) | 1.0 (1.3) | 2.5 (1.4) | 0.4 (0.4) |
|  | |  |  |  |  |
| Scanned region | |  |  |  |  |
| Brain, n (%) | | | 199 (100.0) | 59 (29.6) | 140 (70.4) |
| Spinal cord, n (%) | | | 138 (69.3) | 34 (24.6) | 104 (75.4) |
|  | | |  |  |  |
| Gadolinium-based agent administered, | |  |  |  |  |
| Yes, n (%) | | | 166 (83.4) | 49 (29.5) | 117 (70.5) |
| No, n (%) | | | 33 (16.6) | 10 (30.3) | 23 (69.7) |
|  | | |  |  |  |
| Gadolinium-enhancing lesions, local assessment | |  |  |  |  |
| Brain | | N of patients (missing) | 164 (35) | 47 (12) | 117 (23) |
|  |  | Mean (SD) | 0.8 (2.2) | 0.5 (1.5) | 0.9 (2.4) |
| Spinal Cord | | N of patients (missing) | 117 (82) | 27 (32) | 90 (50) |
|  |  | Mean (SD) | 0.4 (1.3) | 0.1 (0.3) | 0.5 (1.4) |
|  | |  |  |  |  |
| T2 brain lesion count | |  |  |  |  |
| N(missing) | | | 193 (6) | 57 (2) | 136 (4) |
| 0 T2 brain lesions, n (%) | | | 2 (1.0) | 1 (50.0) | 1 (50.0) |
| 1-2 T2 brain lesions, n (%) | | | 10 (5.2) | 2 (20.0) | 8 (80.0) |
| 3-8 T2 brain lesions, n (%) | | | 65 (33.7) | 18 (27.7) | 47 (72.3) |
| ≥ 9 T2 brain lesions, n (%) | | | 116 (60.1) | 36 (31.0) | 80 (69.0) |
|  | | |  |  |  |
| New/enlarged T2 brain lesions compared to the previous scan | | |  |  |  |
| Yes, n (unknown) | | | 33 (8) | 15 (3) | 18 (5) |
| Mean (SD) | | | 3.0 (4.2) | 2.6 (2.5) | 3.5 (5.4) |
|  | | |  |  |  |
| T2 spinal cord lesion count | |  |  |  |  |
| N(missing) | | | 138 (61) | 34 (25) | 104 (36) |
| 0 T2 spinal cord lesions, n (%) | | | 16 (8.0) | 4 (25.0) | 12 (75.0) |
| 1-2 T2 spinal cord lesions, n (%) | | | 41 (20.6) | 11 (26.8) | 30(73.2) |
| 3-8 T2 spinal cord lesions, n (%) | | | 81 (40.7) | 19 (23.5) | 62 (76.5) |
|  | | |  |  |  |
| New/enlarged T2 spinal cord lesions compared to the previous scan | | |  |  |  |
| Yes, n (unknown) | | | 13 (6) | 6 (2) | 7 (4) |
| Mean (SD) | | | 1.4 (1.0) | 1.5 (1.3) | 1.3 (0.6) |
|  | | |  |  |  |
| CRC data | | |  |  |  |
| T2 brain lesion volume (ml) | N of patients (missing) | | 173 (26) | 53 (6) | 120 (20) |
|  | Mean (SD) | | 4.64 (5.53) | 3.08 (2.62) | 5.32 (6.30) |
|  |  | |  |  |  |
| Gadolinium-enhancing lesions | N of patients (missing) | | 152 (47) | 50 (9) | 102 (38) |
|  | Mean (SD) | | 0.5 (2.0) | 0.4 (1.5) | 0.6 (2.3) |
|  |  | |  |  |  |
| T1-hypointense lesion volume (ml) | N of patients (missing) | | 107 (92) | 30 (29) | 77 (63) |
|  | Mean (SD) | | 1.14 (2.05) | 0.41 (0.67) | 1.41 (2.33) |

Values are expressed as n (%) and mean (SD).

*Abbreviations: SD, standard deviation; CRC, Central Reading Center.*
